# Phosphatidylinositol signalling defects implicated in IBD

**Published:** 2014-01

**Figure:**
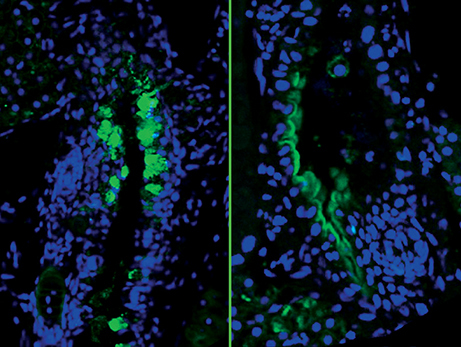


Inflammatory bowel disease (IBD), the collective term for a group of chronic conditions characterised by inflammation of the gastrointestinal tract (including Crohn’s disease and ulcerative colitis), affects an estimated 1.4 million individuals in the US, and its incidence is increasing worldwide. Current treatments generally manage the symptoms rather than provide a cure, highlighting the need to develop effective molecular therapies. In this new study, Nathan Bahary and colleagues use zebrafish to demonstrate that a deficiency in phosphatidylinositol (PI) signalling could be involved in the pathogenesis of IBD. Abnormalities in PI signalling have been linked with gastrointestinal disorders; however, the mechanisms remained unclear. Here, the authors show that PI-synthesis-deficient zebrafish display persistent ER stress and disrupted intestinal architecture, resulting in IBD-like pathologies. These effects can be mimicked by pharmacological induction of ER stress, whereas abrogation of ER stress using chemical chaperones rescues the disease phenotype. These findings link PI signalling defects with ER-stress-mediated gastrointestinal disease, revealing a mechanism that could be targeted in the development of curative therapies for IBD. Page 93

